# Stimulative role of ST6GALNAC1 in proliferation, migration and invasion of ovarian cancer stem cells via the Akt signaling pathway

**DOI:** 10.1186/s12935-019-0780-7

**Published:** 2019-04-05

**Authors:** Wen-Yan Wang, Yun-Xia Cao, Xiao Zhou, Bing Wei, Lei Zhan, Shi-Ying Sun

**Affiliations:** 1 Department of Obstetrics and Gynecology, The Second Hospital of Anhui Medical University, Hefei, 230601 People’s Republic of China; 2 Teaching and Research Group of Obstetrics & Gynecology, Anhui Medical University, No. 81, Meishan Road, Hefei, 230032 Anhui People’s Republic of China; 3 Department of Cardiothoracic Surgery, The Second Hospital of Anhui Medical University, Hefei, 230601 People’s Republic of China

**Keywords:** Ovarian cancer, ST6GALNAC1, Akt signaling pathway, Ovarian cancer stem cells, Proliferation, Migration, Invasion, Tumorigenicity, Tumorsphere

## Abstract

**Background:**

Ovarian cancer is known as one of the most common cancers in the world among women. ST6GALNAC1 is highly expressed in cancer stem cells (CSCs), which correlates to high tumor-initiating, self-renewal and differentiation abilities. This present study aims to investigate how ST6GALNAC1 affects ovarian cancer stem cells (OCSCs).

**Methods:**

In order to identify the differentially expressed genes related to ovarian cancer, microarray-based gene expression profiling of ovarian cancer was used, and ST6GALANC1 was one of the identified targets. After that, levels of ST6GALNAC1 in OCSCs and ovarian cancer cells were examined. Subsequently, an Akt signaling pathway inhibitor LY294002 was introduced into the cluster of differentiation 90^+^ (CD90^+^) stem cells, and cell proliferation, migration and invasion, levels of CXCL16, EGFR, CD44, Nanog and Oct4, as well as tumorigenicity of OCSCs were examined.

**Results:**

By using a comprehensive microarray analysis, it was determined that ST6GALNAC1 was highly expressed in ovarian cancer and it regulated the Akt signaling pathway. High levels of ST6GALNAC1 were observed in OCSCs and ovarian cancer cells. Silencing ST6GALNAC1 was shown to be able to reduce cell proliferation, migration, invasion, self-renewal ability, tumorigenicity of OCSCs. In accordance with these results, the effects of ST6GALNAC1 in OCSCs were dependent on the Akt signaling pathway.

**Conclusions:**

When taken together, our findings defined the potential stimulative roles of ST6GALNAC1 in ovarian cancer and OCSCs, which relied on the Akt signaling pathway.

## Background

Ovarian cancer has been reported to account for nearly 2.5% of female malignancies and 5% of deaths related to female cancers. This is caused by poor survival rates mostly owing to diagnosis at the advanced phase [1]. Ovarian cancer had long been a complicated and malignant tumor that is often difficult to diagnose and cure [2]. More than 90% of malignant ovarian tumors are carcinomas, which may originate from the surface epithelium and/or fallopian tube of the ovary, with a poor prognosis and an overall 5-year survival rate of less than 40% [3].

Cancer stem-like cells (CSCs), or cancer-initiating cells (CICs), are highly tumorigenic cancer cells, and often possess the abilities of self-renewal, differentiation and tumorigenesis [4]. CSCs were once thought to be the causes of phenotypic and functional heterogeneities of tumors, and also considered one of the reasons for chemotherapy resistance and eventual tumor recurrence [5]. Treatment for those patients that were in the late stages of ovarian cancer included cytoreductive surgery and platinum based cytotoxic chemotherapy, but both methods were considered failures since most patients relapsed [6]. Therefore, it is urgent to identify novel therapeutic and diagnostic targets to help improve the prognosis and treatment of ovarian cancer patients.

ST6GALNAC1 encodes a sialyltransferase that acts as a catalyst for the synthesis of cancer-related sialyl-Tn (sTn) antigen critical for cell mobility [7]. It has been reported that ST6GALNAC1 was overexpressed in gastric, breast and prostate cancer cell lines, inducing sTn expression [8–11]. Previous findings have shown that ST6GALNAC1 played a part in elevating CSC phenotypes of colorectal cancer by activating the Akt signaling pathway [4]. The Akt signaling pathway is an essential intracellular pathway that can regulate multiple cellular processes including cell homeostasis, proliferation, survival, migration and differentiation. [12]. The Akt signaling pathway is activated in nearly 70% of ovarian cancers, leading to hyperactive signaling transmission that promotes cell growth, proliferation, and angiogenesis [13]. It is highly possible that ST6GALNAC1 may affect biological characteristics of ovarian cancer stem cells (OCSCs) via the Akt signaling pathway. Therefore, in this present study, the aim was to investigate the role of ST6GALNAC1 in the occurrence and development of ovarian cancer and whether ST6GALNAC1 functioned through the Akt signaling pathway.

## Materials and methods

### Ethics statement

All experiments were approved by the Ethics Committee and Experimental Animal Ethics Committee of the Second Hospital of Anhui Medical University. Written informed consent was obtained from each participant. The tissue specimens of 48 patients with ovarian cancer were collected strictly in accordance with the specimen collection standard during the operation. All efforts were made to minimize suffering.

### Bioinformatics prediction of molecular mechanism of ovarian cancer

Ovarian cancer gene expression chips were searched in the gene expression omnibus (GEO) database (https://www.ncbi.nlm.nih.gov/geo/). GSE14407, GSE18520, GSE38666 and GSE66957 chips were used to screen the differentially expressed genes in ovarian cancer. The detailed information of the chips used is shown in Table 1. The R-language affinity package was used for background correction and standardized pretreatment of gene expression data, and the limma package was used to screen the differentially expressed genes. The correct *p* value was expressed via *adj.P.Va.l*;|log2 FC| > 3.0 and *p* value < 0.05 were set as the threshold to screen out differentially expressed genes. The differentially expressed genes of the four gene chips were analyzed by jvenn (http://JVenn.tour.inra.fr/app/example.htmL). By using the Chilibot (http://www.childbot.net/index.htmL) the relationship between differentially expressed genes and ovarian cancer was investigated. The DisGeNET gene-disease related database (http://www.disgenet.org/web/DisGeNET/menu/search?4) was used to screen out ovarian cancer-related genes. The differentially expressed genes and ovarian cancer related genes were introduced into String database (https://string-db.org/), and the gene function analysis and an interaction analysis were carried out. The gene interaction network was visualized by Cytoscape 3.6.0 software [14].

**Table 1:** Information of ovarian cancer gene chips

Accession	Platform	Organism	Sample
GSE14407	GPL570	Homo sapiens	12 healthy ovarian surface epithelia samples and 12 serous ovarian cancer epithelia samples
GSE18520	GPL570	Homo sapiens	53 papillary serous ovarian adenocarcinoma tumor specimens and 10 normal ovarian surface epitheliums
GSE38666	GPL570	Homo sapiens	12 normal ovarian surface epithelium (OSE) and 18 ovarian cancer epitheliums
GSE66957	GPL570	Homo sapiens	57 ovarian carcinomas and 12 ovarian normal samples

### Cell and tissue samples

Five ovarian cancer cell lines (SKOV3, CAOV3, HO8910, PEO4, A2780) and one normal ovarian cell line (IOSE80) were all purchased from Shanghai Institute of Cell Biology, Chinese Academy of Science.

In total, 48 cases of ovarian cancer tissues were collected, which were received from the patients that underwent surgery for ovarian cancer in the Second Hospital of Anhui Medical University. The tissues were frozen at − 80 °C, dehydrated by an automatic dehydrator, and then embedded and preserved in paraffin.

### Sorting and identification of OCSCs

In order to identify and sort ovarian cancer stem cells, an immunomagnetic bead sorting was used. SKOV3 cells in logarithmic growth phase were collected and washed with 300 μL phosphate buffer saline (PBS) buffer (0.5% bovine serum albumin (BSA), 2 mM ethylenediaminetetraacetic acid (EDTA), 0.01% PBS). A total of 100 μL FCR blocker and 100 μL clusters of differentiation 90^+^ (CD90^+^) magnetic micro-beads were added, and the cells were incubated in dark at 4 °C for 30 min after being fully suspended. The cells were then washed with PBS 10 to 20 times and centrifuged at 179*g* for 5 min. The rinsing buffer was removed and 500 μL PBS was added, with the cell suspension being transferred into the MS sorting column installed in the magnetic sorting rack with a Pasteur tube. After the cell suspension was removed, the cells (CD90^−^) were removed from the sorting column with buffer of 4 times volume, and then collected. After the buffer solution was removed, 1 mL buffer solution was added, the sorting column was removed from the magnetic sorting rack, and the buffer solution (containing CD90^+^ cells) was pumped into a collecting bottle by a plug matched with the sorting column. Parts of the sorted CD90^+^ stem cells were inoculated into a 100 mL culture flask and incubated with 10 mL Dulbecco’s modified eagle medium (DMEM)/F12 (1:1) CO_2_ culture medium (containing 20 μg/L EGF, 20 μg/L bFCF and 20 μg/L LIF). The medium was changed every 4 days. The remaining CD90^+^ cells and the CD90^−^ cells were cultured in RPMI1640 serum-free medium (SFM) in a 5% CO_2_ 37 °C incubator respectively. Cell morphology and tumor sphere formation of CD90^+^ stem cells were observed every day.

In identifying OCSCs, reverse transcription quantitative polymerase chain reaction (RT-qPCR) and western blot analysis were used in order to detect the expression of stem cell related genes CD44, Nanog, and Oct4. Glyceraldehyde 3-phosphate dehydrogenase (GAPDH) was used as the internal reference gene, and the relative expression of the gene was represented as 2^−ΔΔCt^. The cancer stem cells were enriched through tumor sphere formation experiments.

### Cell grouping and transfection

Lentivirus vectors were used to package three pairs of si-ST6GALNAC1 (si-1 [CGAGUUUACAGUUGUGAAAUC], si-2 [GGAGCAGUGUCAACAAGGACG], si-3 [GGCUCAUUGUUAAGACAAAGG]), and overexpressed plasmid (ST6GALNAC1). Empty vector si-NC and PCDNA3.0 were taken as the silencing and overexpressing controls. After that, cells were treated based on the instructions of lip2000 and si-3 with the best silencing effects was selected for subsequent experiment.

The collected OCSCs were randomly assigned into eight groups: the si-NC (cells infected with silent blank plasmid), si-ST6GALNAC1 (cells infected with silent ST6GALNAC1 plasmid), empty vector (cells infected with empty vector PCDNA3.0), ST6GALNAC1 (cells infected with ST6GALNAC1 plasmid), dimethyl sulfoxide (DMSO) (cells treated with DMSO), LY294002 (cells treated with Akt signal pathway inhibitor LY294002), ST6GALNAC1 + DMSO and ST6GALNAC1 + LY294002 groups, respectively. Cells were inoculated into the 6-well plate 24 h prior to treatment. When cell confluence reached about 50%, OCSCs were treated instantly via lipofectamine 2000 (Invitrogen, Carlsbad, California, USA). After 6 h of treatment, the culture medium was replaced and OCSCs continued to be cultured for 48 h and then collected for subsequent experiment.

### RT-qPCR

TRIzol (Invitrogen, Carlsbad, California, USA) was used in order to extract the total RNA from tissues and cells. Primer sequences for RT-qPCR are shown in Table 2. The reaction conditions were pre-denaturation at 95 °C for 10 min, 40 cycles of denaturation at 95 °C for 10 s, annealing at 60 °C for 20 s and extension at 72 °C for 34 s. GAPDH served as the internal reference. The relative expression of genes was calculated as 2^−ΔΔCt^. Each experiment was repeated three times.

**Table 2:** Primer Sequences for RT-qPCR

Gene	Primer sequence
ST6GALNAC1	F: 5′-CGAAATAGGAGGCCTTCAGA-3′
	R: 5′-AGAGAGTGAGGTTGGGCAGA-3′
CD44	F: 5′-AGAAGGTGTGGGCAGAAGAA-3′
	R: 5′-AAATGCACCATTTCCTGAGA-3′
Nanog	F: 5′-TGCAAATGTCTTCTGCTGAGAT-3′
	R: 5′-GTTCAGGATGTTGGAGAGTTC-3′
Oct4	F: 5′-CAGAAGGGCAAGCGATCAAG-3′
	R: 5′-GGGCCAGAGGAAAGGACACT-3′
CXCL16	F: 5′-CGAGCTCAAGCTTCGAATTCTGATGTCTGGGAGTCAGAGCG-3′
	R: 5′-TGGTGGCGACCGGTGGATCCCGGGTATTAGAGTCAGGTGCCAC-3′
EGFR	F: 5′-GTCTGCCATGCCTTGTGCTC-3′
	R: 5′-CTTGTCCACGCATTCCCTGC-3′
GAPDH	F: 5′-GAAGGTGAAGGTCGGAGTC-3′
	R: 5′-GAAGATGGTGATGGGATTTC-3′

RT-qPCR, reverse transcription quantitative polymerase chain reaction; CD44, cluster of differentiation 44; Oct4, octamer-binding transcription factor 4; CXCL16, CXC chemokine ligand 16; EGFR, epidermal growth factor receptor; GAPDH, glyceraldehyde 3-phosphate dehydrogenase

### Western blot analysis

The cells were lysed in RIPA Lysis Buffer (BB-3209, Best Bio biotechnology Company, Shanghai, China) and the cell lysates were separated by Sodium Dodecyl Sulfate Polyacrylamide Gel Electrophoresis (SDS-PAGE) gel electrophoresis, which was then transferred onto the polyvinylidene fluoride (PVDF) membrane. The membrane was incubated at 4 °C overnight with primary polyclonal rabbit antibodies to ST6GALNAC1 (ab82821, 1:1000), Oct4 (ab18976, 1:1000), CXCL16 (ab101404, 1:2000), p-PI3K (ab182651, 1:2000), and CD44 (ab157107, 1:2000), and primary monoclonal antibodies to PI3K (ab151549, 1:1000), Akt (ab179463, 1:1000), p-Akt (ab192623, 1:1000), Nanog (ab109250, 1:1000), S6 (ab32529, 1:1000), p-S6 (ab109393; 1:1000), epidermal growth factor receptor (EGFR) (ab52894, 1:1000), β-catenin (ab32572, 1:5000), and GAPDH (ab181603, 1:5000), respectively. These antibodies were all from Abcam Inc., (Cambridge, MA, USA). The membrane was then incubated with horseradish peroxidase (HRP) goat anti-rabbit (A2012, ABBkine, California, USA) at 37 °C for 1 h. GAPDH was used as an internal control. Relative level of target protein was shown to be equal to the gray value of target protein band/gray value of GAPDH band from the same sample. Each experiment was repeated three times.

### EdU assay

Cells in logarithmic phase were inoculated into 96-well plate with 4 × 10^3^–1 × 10^5^ cells per well and cultured to normal growth stage. EdU solution was diluted with cell culture medium to the final concentration of 50 μM. Each well was incubated with 100 μL 50 μM EdU culture medium for 2 h. The cells were then washed with PBS 1 to 2 times, 5 min each time. Each well was incubated at room temperature with 50 μL cell fixing solution (PBS containing 4% paraformaldehyde) for 30 min and then incubated with 50 μL 2 mg/mL glycine for 5 min. Following incubation, cells were incubated with 100 μL penetrant (0.5% Triton X-100 PBS) in each well for 10 min. Next, 100 μL of 1X Apollo dyeing reaction solution was added to each well and reacted for 30 min in a dark room which was kept at room temperature, following which, cells were washed with 100 μL penetrant 2 to 3 times, 10 min each time. After that, each well was washed 1 to 2 times with 100 μL methanol for 5 min each time, and then washed in PBS once for 5 min. Reagent F was diluted with deionized water at a ratio of 100:1. A total of 100 μL 1× Hoechst33342 reaction solution was added into each well, and reacted for 30 min in a dark room which was kept at room temperature. Images were obtained under a fluorescence microscope. The numbers of cells labeled with (red) or without (blue) were calculated. The rate of EdU positive cells = the number of cells labeled with EdU/(the number of cells labeled with EdU + the number of unlabeled cells) × 100%.

### Transwell assay

In order to conduct and understand the results of the Transwell assay, cell migration was investigated. When cell confluence reached 80%, cells were starved in the serum-free Dulbecco’s Modified Eagle Medium (DMEM) for 24 h. The lower chambers of the Transwell (Corning, New York State, America) were pre-incubated with serum-free DMEM at 37 °C for 1 h. The cells were digested, resuspended in serum-free DMEM, and diluted to 3 × 10^5^ cells/mL, with 100 μL cells then being added to the apical chamber, and 600 μL of 10% serum DMEM medium being added to the basolateral chamber (serum was used as chemotaxis factor). After incubation for 24 h, cells on the upper surface of the Transwell chamber were removed. The chamber was washed with phosphate buffer saline (PBS), soaked in pre-cooled methanol for 30 min in order to fix the cells that reached the bottom of the chamber, which were then stained with 0.1% crystal violet solution for 10 min. A total of 6 fields were selected, and images were acquired under an inverted microscope (Olympus, Tokyo, Japan). The cells were counted afterwards and the experiment was repeated 3 times.

The second part of conducting Transwell assay was to investigate cell invasion. Matrigel was diluted in serum-free DMEM at the ratio of 1:10. The concentration of inoculated cells was adjusted to 1.0 × 10^5^ cells/mL, and the remaining operations were the same with those in the cell migration experiment. The experiment was repeated 5 times.

### Tumorsphere formation

A total of 1 × 10^4^ CD90^+^ OCSCs were inoculated into low-adsorption 96-well plate, cultured in serum-free DMEM-F12 medium containing 20 ng/mL epidermal growth factor (EGF) and 20 ng/mL transforming growth factor-β (FGF-β), with one semi-quantitative change of liquid being conducted every 2 days. After consistent culture for a period of 10 days, the microspheres were filtered using 70 μM filter membrane with the filtration abandoned, and the rest of the microspheres that were less than 70 μM were counted [15].

### Colony formation in soft agar

A total of 0.7% low melting point agarose was prepared using fresh DMEM medium and 2 mL agarose was plated onto a 100 mm diameter culture dish. Cell suspension (1 mL) was mixed with 1 mL 0.7% agarose solution and inoculated gently to the dish after the agarose was solidified, with three replicates being prepared. The cells were cultured at 37 °C with 5% CO_2_ for 1 month with cell medium being changed once every 2 to 3 days. The cells were then counted under an inverted microscope. Only the masses with more than 50 cells were judged as a cell colony, and images were taken.

### In vivo limiting dilution assay

Cells were inoculated to a low adherent culture plate. After 7 days of culture, OCSC spheres were collected into a 10 mL glass centrifuge tube, centrifuged, and the supernatant was discarded. The cells were then washed once with saline. A total of 1 mL 0.5% trypsin was added into the incubator at 37 °C. The bottom of the plate was lightly flipped every 2 min. The cells were digested for 10 min in order to make single cells. The culture was terminated by adding 3 mL complete medium, with the cells then being centrifuged in order to collect cell sedimentation. The cells were then washed once with saline. The cells were added with an appropriate amount of saline, and then gently pipetted into single cell suspension to perform cell counting. Different numbers of cells (1 × 10^3^, 5 × 10^3^, 1 × 10^4^, and 5 × 10^4^) were respectively re-suspended in 50 μL of saline, and then mixed with 50 μL Matrigel Matrix (1:1), and subcutaneously inoculated to NOD-SCID mice. Two weeks following inoculation, tumor formation was observed and recorded. The ratio of cancer stem cells was calculated using the ELDA software (http://bioinf.wehi.edu.au/software/elda/index.htmL) [16].

### Tumorigenesis in nude mice

Cells were inoculated to a low adherent culture plate. Following 7 days of culture, OCSC spheres were collected into a 10 mL glass centrifuge tube, centrifuged, and the supernatant was discarded. The spheres were washed once with saline and a total of 1 mL 0.5% trypsin was added into the incubator at 37 °C with the bottom being lightly flipped every 2 min. After treatment for 10 min, the cell sphere was decomposed into single cells. The cells were then added with 3 mL complete medium to terminate the culture and centrifuged to collect cell sedimentation, and the cells were washed once with saline. An appropriate amount of saline was added with the cells gently pipetted into single cell suspension to perform cell counting. A total of 2 × 10^6^ cells were re-suspended in 50 μL of saline, mixed with 50 μL Matrigel Matrix (1:1). A total of 36 NOD-SCID mice were divided into si-NC group (NOD-SCID mice inoculated with OC stem cells with silent empty plasmid), si-ST6GALNAC1 (NOD-SCID mice inoculated with OCSCs with silent ST6GALNAC1 plasmid), empty vector group (NOD-SCID mice inoculated with OCSCs with silent empty plasmid), ST6GALNAC1 group (NOD-SCID mice inoculated with overexpressed OCSCs with plasmid), ST6GALNAC1 + DMSO group (NOD-SCID mice inoculated with overexpressed OCSCs with plasmid and DMSO cancer stem cells) and ST6GALNAC1 + LY294002 group (NOD-SCID mice inoculated with overexpressed OCSCs with plasmid and injected with OCSCs with Akt signaling pathway inhibitor). Each group contained 6 samples. The cells were subcutaneously inoculated to NOD-SCID mice. Two weeks after inoculation, tumor volumes were recorded.

### Immunohistochemical staining

β-Catenin (CST, 9562S) was subjected to immunohistochemical staining using the immunohistochemistry kits (Key-GEN, Nanjing, China). Tumor tissues collected from the euthanized mice were fixed with formalin and embedded with paraffin. The paraffin-embedded tissue sections were then deparaffinized, hydrated, washed in 1% PBST, and blocked with 5% BSA at 37 °C for 1 h. The slides were then incubated overnight at 4 °C with the antibody in PBS containing 5% BSA and then incubated with the addition of biotinylated secondary antibody at room temperature for 1 h. The slides were then cultured with streptavidin–horseradish–peroxidase, stained using diaminobenzidine (DAB) substrate, and counterstained using hematoxylin. After that, the tissues were observed under the inverted microscope (Olympus, Center Valley, PA, USA).

### Statistical analysis

Statistical analyses were conducted using SPSS 21.0 (IBM-SPSS, Chicago, Illinois, USA). Each experiment was carried out at least three times. The measurement data were expressed by mean ± standard deviation. The data were consistent with the normal distribution and the variance was uniform. Expression of ST6GALNAC1 in cancer tissues and paracancerous tissues was analyzed using paired *t* test. The data between the other two groups were examined by the non-paired *t* test (independent sample *t* test). Kolmogorov–Smirnov method was applied for data normality test, and data among multiple groups with a normal distribution was compared using one-way AVONA, in which Tukey’s post hoc test was used in multiple comparisons. Comparisons of proliferation ability at different time points in the experiment process were analyzed by repeated measurement ANOVA. A *p* value < 0.05 was indicative of significant statistical difference.

## Results

### ST6GALNAC1 is highly expressed and may regulate the Akt signaling pathway in ovarian cancer

**Fig. 1:**
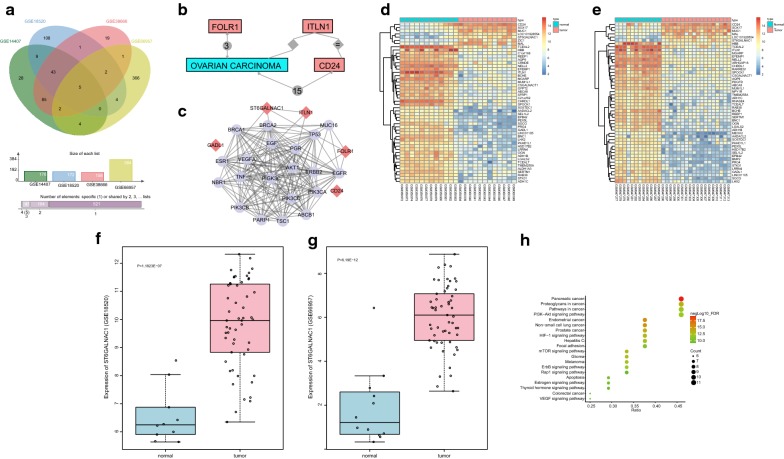
ST6GALNAC1 is highly expressed in ovarian cancer and mediates the Akt signaling pathway. **a** Comparison of the differentially expressed genes in GSE14407, GSE18520, GSE38666, and GSE66957, and 5 intersecting differentially expressed genes; **b** the relationship between ITLN1, GADL1, CD24, FOLR1, ST6GALNAC1, and ovarian cancer. Relationship between GADL1 and ST6GALNAC1 and ovarian cancer is not shown in the figure; **c** the interaction network of differentially expressed genes and disease genes in ovarian cancer, red diamonds represented differentially expressed genes, and purple circles represented disease genes; **d**, **e** the heat maps of the first 50 differentially expressed genes in GSE14407 and GSE38666. The abscissa indicated the sample number, the ordinate indicated the differentially expressed genes, and the upper right histogram was the color scale. Each rectangle corresponded to a sample expression value; **f**, **g** the expression changes of ST6GALNAC1 in GSE18520 and GSE66957; **h** KEGG enrichment analysis of ovarian cancer differentially expressed genes and disease genes

R language was used to screen differentially expressed genes from the ovarian cancer gene chips, and 176, 172, 158, and 384 differentially expressed genes were screened out respectively from GSE14407, GSE18520, GSE38666, and GSE66957 microarrays based on|log2 FC| > 3.0 and *p* value < 0.05. The differentially expressed genes in the four chips were compared and Venn maps of the genes were constructed (Fig. 1a). A total of five intersection genes (intelectin 1 (ITLN1), glutamate decarboxylase (GADL1), cluster of differentiation 24 (CD24), folate receptor (FOLR1), and ST6GALNAC1) were identified and subjected to follow-up analysis. The relationships between ITLN1, GADL1, CD24, FOLR1, ST6GALNAC1 and OVARIAN CARCINOMA were analyzed in Chilibot database (Fig. 1b). There is no evidence that GADL1 and ST6GALNAC1 are associated with ovarian cancer in this database, indicating that there are few studies on effects of GADL1 and ST6GALNAC1 on ovarian cancer. The genes related to ovarian cancer were searched in the DisGeNET Database, and the top 20 genes were selected as disease genes. An interaction network of ovarian cancer related differentially expressed genes and disease genes (Fig. 1c) was established. The most relevant differential genes in this network were FOLR1 and ST6GALNAC1, suggesting that these two genes may affect ovarian cancer. Previous studies have already shown that abnormal expression of FLOR1 was relevant to ovarian cancer [17, 18]. There are few studies on the effects that ST6GALNAC1 has on ovarian cancer; therefore, the differential expression of ST6GALNAC1 in ovarian cancer and possible molecular mechanisms were investigated in this present study. The heat maps of the top 50 differentially expressed genes in GSE14407 and GSE38666 are shown in Fig. 1d, e respectively. The expression of ST6GALNAC1 in ovarian cancer tissues was significantly higher than that in normal tissues. The expression profile of ST6GALNAC1 in GSE18520 and GSE66957 chips was extracted and ST6GALNAC1 was abnormally highly-expressed in ovarian cancer in GSE18520 (Fig. 1f) and GSE66957 (Fig. 1g) chips. The Kyoto Encyclopedia of Genes and Genomes (KEGG) enrichment analysis was performed on the ovarian cancer differentially expressed genes and disease genes in the String database. The results are shown in Fig. 1h. Combined with the gene interaction network information, it was found that FOLR1 and ST6GALNAC1 were enriched in the PI3K-Akt signaling pathway, and ST6GALNAC1 could activate the Akt pathway. Therefore, it was speculated that ST6GALNAC1 was abnormally expressed in ovarian cancer and may mediate the Akt signaling pathway.

### ST6GALNAC1 is expressed at high levels in OCSCs and ovarian cancer cells

**Fig. 2:**
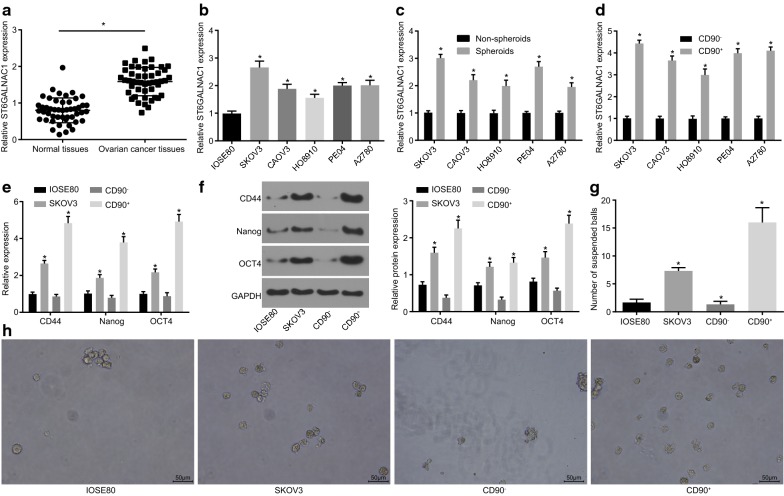
ST6GALNAC1 is highly expressed in OCSCs and ovarian cancer cells. **a** Relative expression of ST6GALNAC1 in ovarian cancer (n = 48; data were compared using paired *t*-test; *p < 0.05 vs. the normal tissues); **b** relative expression of ST6GALNAC1 in ovarian cancer cell line and ovarian cell line (*p < 0.05 vs. IOSE80); **c** relative expression of ST6GALNAC1 in non-spheroids and spheroids (*p < 0.05 vs. non-spheroids); **d** relative expression of ST6GALNAC1 in CD90^+^ and CD90^−^ (*p < 0.05 vs. CD90^−^); **e** RT-qPCR of CD44, Nanog, and Oct4 (*p < 0.05 vs. IOSE80); **f** Western blot analysis of CD44, Nanog, and Oct4; **g** tumorsphere formation number (*p < 0.05 vs. IOSE80); **h** the tumorsphere formation 10 days after culture. The measurement data were expressed as mean ± standard deviation. The test between two groups was analyzed by *t*-test, and the one-way ANOVA was used for analysis among the multiple groups. The experiment was repeated 3 times

RT-qPCR and western blot analysis were performed in order to investigate the expression of ST6GALNAC1 on ovarian cancer tissues and paracancerous tissues from 48 different ovarian cancer patients. RT-qPCR results exhibited that the expression of ST6GALNAC1 was higher in cancer tissues than that in paracancerous tissues (Fig. 2a). The expression of ST6GALNAC1 in several ovarian cancer cells was further examined. When compared with IOSE80, the expression of ST6GALNAC1 in SKOV3, CAOV3, HO8910, PE04, and A2780 was all increased, with that of SKOV3 being the highest (Fig. 2b).

The expression of ST6GALNAC1 was then further verified in OCSCs isolated from SKOV3 cells by RT-qPCR. When compared with non-spheroids, the expression of ST6GALNAC1 was elevated in CSCs spheroids (Fig. 2c). CD90 was a commonly used in CSCs markers, so immunomagnetic beads were used to sort CD90 positive and negative cells from SKOV3, CAOV3, HO8910, PE04, and A2780 cells. The expression of ST6GALNAC1 was first detected. When compared to that of CD90− cells, the expression of ST6GALNAC1 was increased in the subgroup of CD90^+^ stem cells, with the highest expression from the SKOV3 cell line (Fig. 2d). RT-qPCR and western blot analysis (Fig. 2e, f) were used in order to verify the expression of stem cell-related gene (CD44, Nanog, and Oct4) and the abilities of suspension tumorsphere formation (Fig. 2g, h) in those cells. The expression of CD44, Nanog, and Oct4, and suspension tumorsphere numbers in CD90^+^ cells were significantly higher when compared to those of IOSE80, SKOV3, and CD90^−^ cells, indicating that CD90^+^ stem cells had stronger proliferation, differentiation, and self-renewal capacities. Therefore, CD90^+^ stem cells isolated from SKOV3 cells were selected for subsequent experiments. All aforementioned results showed that ST6GALNAC1 was highly expressed in CD90^+^ stem cells and ovarian cancer cells.

### Si-3 has the highest knockout efficacy

**Fig. 3:**
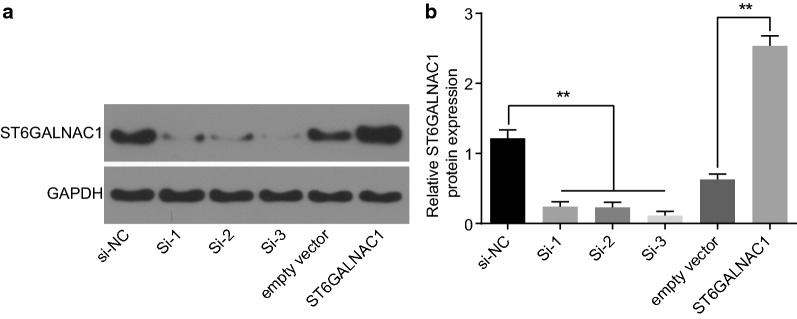
Si-3 exhibits the best knockout efficacy. **a**, **b** Identification and statistical analysis of the efficiency of the siRNA knockout or overexpressing ST6GALNAC1. The data were expressed as mean ± standard deviation and analyzed using t-test. *p < 0.05; **p < 0.01, n = 3

OCSCs were knocked out using three ST6GALNAC1 siRNAs: si-1, si-2, and si-3. Chaotic non-targeted siRNA was used as blank controls. As determined by western blot analysis, si-3 had the highest knockout efficacy reaching 70%. Therefore, si-3 was selected for subsequent experiments. In addition, plasmid overexpressing ST6GALNAC1 was also constructed to treat OSSCs with empty vector pCDNA as controls. Furthermore, the expression of ST6GALNAC1 was detected to be elevated by western blot analysis (Fig. 3a, b).

### Silencing ST6GALNAC1 inhibits the proliferation, migration and invasion of OCSCs

**Fig. 4:**
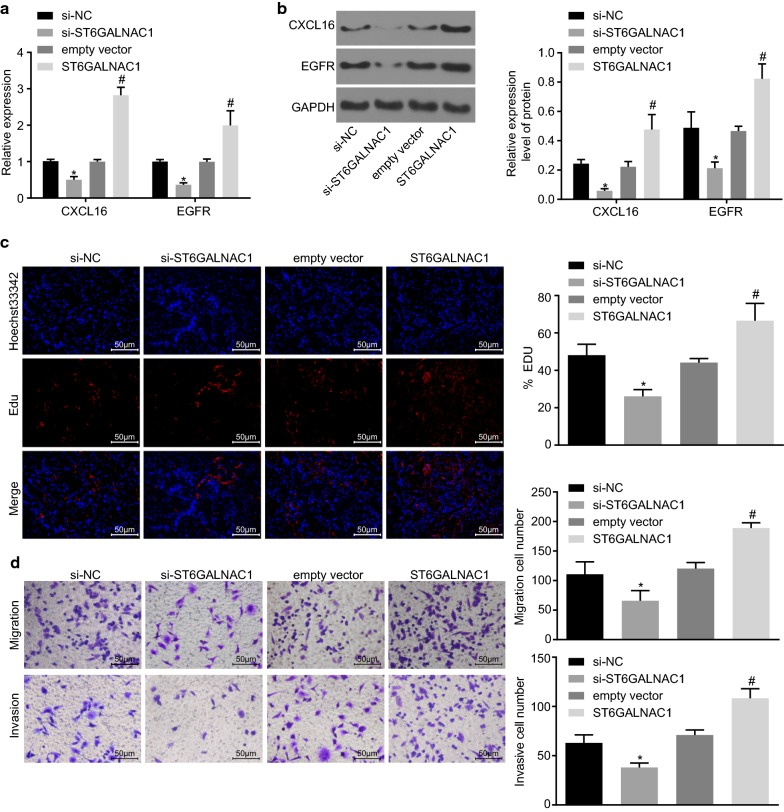
Silencing of ST6GALNAC1 dampens proliferation, migration and invasion of OCSCs. The expression of CXCL16 and EGFR were examined by RT-qPCR (**a**) or western blot analysis (**b**); **c** Proliferation of OCSCs were detected by EdU assay; **d** Migration and invasion of OCSCs were evaluated by Transwell assay. The measurement data were expressed as mean ± standard deviation. The results mentioned above were analyzed by *t*-test. *p < 0.05 vs. the si-NC group; ^#^*p* < 0.05 vs. the empty vector group; the experiment was repeated 3 times

In order to determine the potential role of ST6GALNAC1 in the proliferation, migration and invasion of OCSCs, ST6GALNAC1 was silenced using siRNA or overexpressed in OCSCs and the expression of chemokine CXCL16 and EGFR was detected by RT-qPCR and western blot analysis. As shown in Fig. 4a, b, when compared to that of the si-NC group, the expression of CXCL16 and EGFR in the si-ST6GALNAC1 group displayed an obvious decrease. Conversely, when compared to that of the empty vector group, the expression of CXCL16 and EGFR in the ST6GALNAC1 group was markedly increased. The EdU labeling experiments and Transwell assay showed that the rate of EdU-positive cells and the numbers of cell migration and invasion were significantly decreased in the si-ST6GALNAC1 group when compared with si-NC. Comparatively, the ST6GALNAC1 group exhibited elevated rate of EdU-positive cells and numbers of cell migration and invasion than the empty vector group (Fig. 4c, d). These results indicated that silencing of ST6GALNAC1 suppressed the proliferation, migration, and invasion of OCSCs.

### Silence of ST6GALNAC1 inhibits the self-renewal capacity of OCSCs

**Fig. 5:**
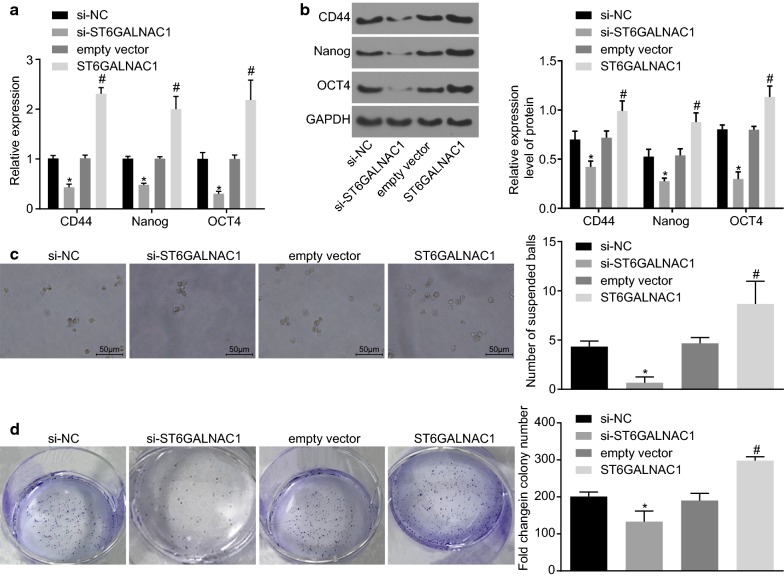
Silence of ST6GALNAC1 suppressed the self-renewal ability of OCSCs. **a** Detection of CD44, Nanog and Oct4 by RT-qPCR; **b** detection of CD44, Nanog and Oct4 by western blot analysis; **c** tumorsphere formation assay was used to test the sphere-forming ability; **d** colony formation in soft agar assay was conducted to examine the colony-forming ability. The measurement data were expressed as mean ± standard deviation. The results mentioned above were analyzed by *t*-test. *p < 0.05 vs. the si-NC group; ^#^*p* < 0.05 vs. the empty vector group; the experiment was repeated 3 times

In order to investigate the effect ST6GALNAC1 had on the self-renewal ability of OCSCs, changes in OCSC surface markers and stem-related transcription factors were examined. RT-qPCR and western blot analysis showed that, when compared to that of the empty vector group, the expression of the surface marker CD44 and the transcription factors Nanog and Oct4 was enhanced in the ST6GALNAC1 group, while the expression of those genes was decreased in the si-ST6GALNAC1 group when compared with the si-NC group (Fig. 5a, b) The effects ST6GALNAC1 had on the tumorsphere-forming and colony-forming abilities of OCSCs were also analyzed. It was found that the number of spheroids formed in the ST6GALNAC1 group was significantly increased, while that in the si-ST6GALNAC1 group was significantly decreased, indicating that overexpression of ST6GALNAC1 promoted the self-renewal of OCSCs (Fig. 5c). Furthermore, colony formation in soft agar suggested that the number of colonies formed in the ST6GALNAC1 group had increased, and that the number of colonies formed in the si-ST6GALNAC1 group was significantly decreased. These results indicated that silence of ST6GALNAC1 inhibited the expansion of OCSCs (Fig. 5d).

### ST6GALNAC1 silence inhibits in vivo tumorigenicity of OCSCs

**Fig. 6:**
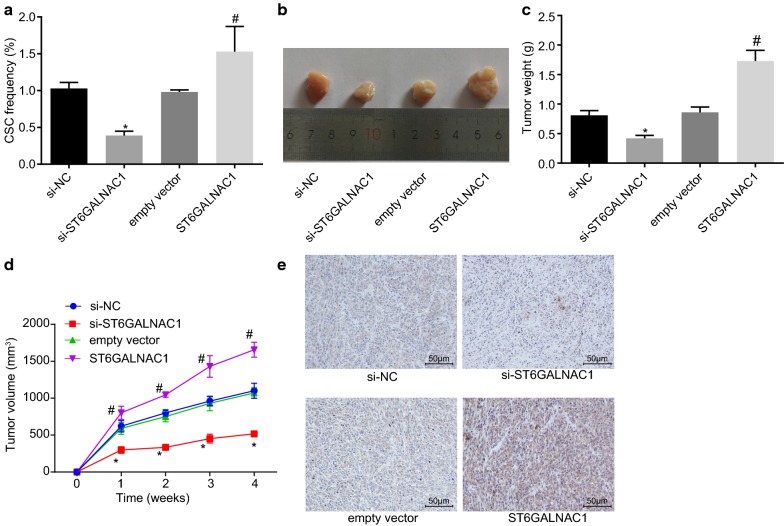
Silence of ST6GAL NAC1 inhibits Akt tumorigenicity of OCSCs in vivo. **a** In vivo limiting dilution assay was used to detect in vivo stem cell ratio of ST6GALNAC1 in mice; **b** the tumor formed by OCSCs in nude mice were examined. **c** Quantification of tumor volumes in **b**; **d** Quantification of tumor weights in **b**; **e** β-catenin staining intensity in OCSCs detected by immunohistochemical staining (×200). The measurement data were expressed as mean ± standard deviation. The in vivo stem cell ratio of ST6GALNAC1 in mice and quantification of tumor volumes were analyzed by *t*-test. The quantification of tumor weights was examined by Two-way ANOVA (n = 6). *p < 0.05 vs. the si-NC group; ^#^*p* < 0.05 vs. the empty vector group; the experiment was repeated 3 times

In order to further analyze the regulatory effect of ST6GALNAC1 on OCSCs in vivo, the tumorigenesis of OCSCs in mice was evaluated. In vivo limiting dilution assay showed that the number of tumors formed in the ST6GALNAC1 group was significantly higher when compared with the empty vector groups, and the proportion of cancer stem cells was also higher (Table 3, Fig. 6a). Furthermore, the effects that ST6GALNAC1 had on tumor growth were further studied by using a mouse subcutaneous tumor model. When compared with the empty vector group, the tumor formation was earlier, the tumor growth was faster, and the tumor volume was larger in the ST6GALNAC1 group, which were all opposite in the si-ST6GALNAC1 group versus the si-NC group (Fig. 6b–d). Moreover, the results of immunohistochemical staining showed that in comparison with the si-NC group, the si-ST6GALNAC1 group exhibited decreased β-catenin staining intensity. However, compared with the empty vector, the β-catenin staining intensity was elevated after overexpressing ST6GALNAC1 (Fig. 6e). These findings revealed that ST6GALNAC1 silencing inhibited tumorigenicity of OCSCs.

**Table 3:** Tumorigenicity of CD90+ spheroid cells

Injected cells	Si-NC	Si-ST6GALNAC1	Empty vector	ST6GALNAC1
1000	0/5	0/5	0/5	1/5
5000	1/5	0/5	1/5	2/5
10,000	2/5	1/5	3/5	4/5
50,000	3/5	2/5	3/5	5/5
Total	6/20	3/20	7/20	12/20

NC, negative control

### The Akt signaling pathway was activated by ST6GALNAC1

**Fig. 7:**
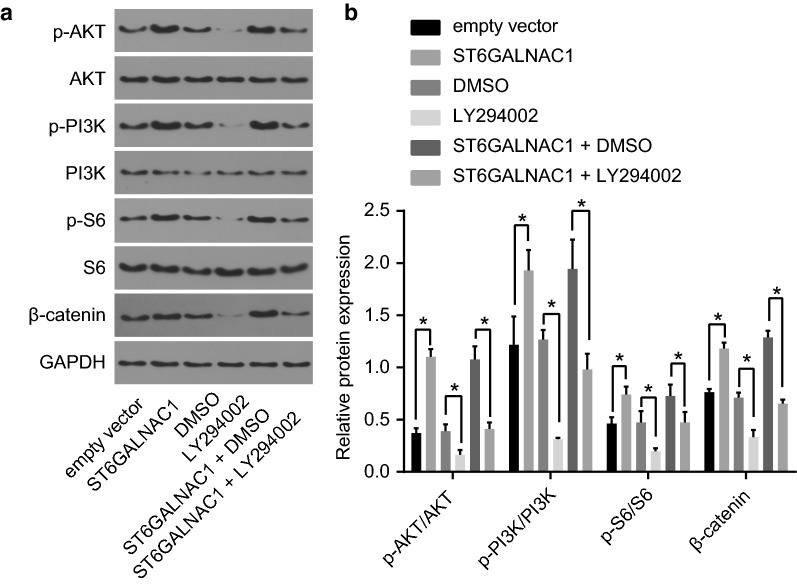
Silencing of ST6GALNAC1 inhibits OCSCs stemness. **a** Protein bands of Akt, p-Akt, PI3K, p-PI3K, S6, p-S6 and β-catenin proteins by western blot analysis; **b** protein expression of Akt, p-Akt, PI3K, S6, p-S6 and β-catenin by western blot analysis; The measurement data were expressed as mean ± standard deviation and analyzed by *t*-test. *p < 0.05; the experiment was repeated 3 times

To investigate the activity of Akt signaling pathway, the levels of β-catenin, phosphorylated PI3K, Akt and S6 were all detected via western blot analysis. When ST6GALNAC1 was overexpressed in OCSCs, the levels of β-catenin and the phosphorylation levels of PI3K, Akt and S6 were also increased, indicating that the Akt signaling pathway was activated. The Akt pathway inhibitor LY294002 was used to further verify the role of the Akt signaling pathway in OCSCs. LY2942002 treatment could block the increase of the levels of β-catenin, and the phosphorylation levels of PI3K, Akt and S6 induced by ST6GALNAC1 overexpression, indicating that suppressing the Akt signaling pathway can inhibit the effect of the ST6GALNAC1 (Fig. 7). These results showed that the Akt signaling pathway was inactivated by silence of ST6GALNAC1.

### ST6GALNAC1 promotes OCSCs stemness through up-regulation of the Akt signaling pathway

**Fig. 8:**
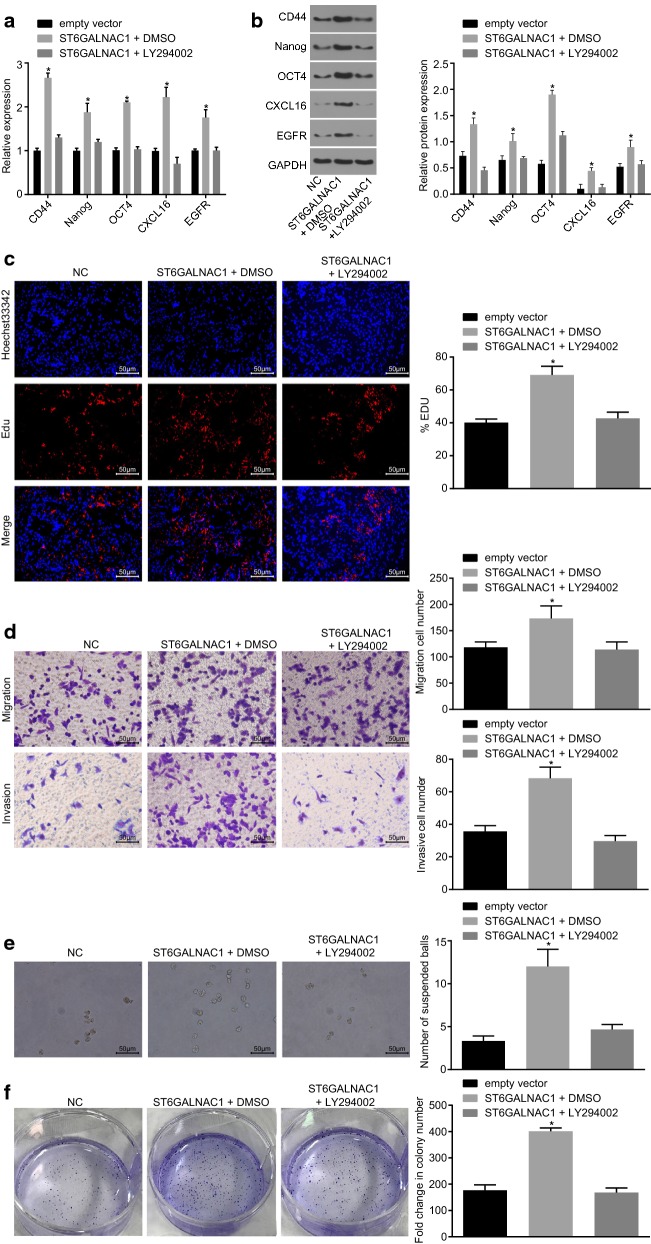
Inactivation of the Akt signaling pathway suppresses proliferation, migration and invasion and characteristics acquisition of OCSCs by ST6GALNAC1. **a** Detection of CD44, Nanog, Oct4, CXCL16 and EGFR by RT-qPCR; **b** detection of CD44, Nanog, Oct4, CXCL16 and EGFR by western blot analysis; **c** detection of stem cell proliferation by EdU assay; **d** detection of stem cell migration and invasion by Transwell assay; **e** tumorsphere formation assay was used to test the ability of different cells to form a sphere after transfection; **f** colony formation in soft agar was used to detect the ability of different cells to form colonies after transfection. The measurement data were analyzed by one-way ANOVA and expressed as mean ± standard deviation. *p < 0.05, compared with the empty vector group; the experiment was repeated 3 times

Since ST6GALNAC1 could activate the Akt signing pathway, the next step was to further investigate whether the Akt signaling pathway is involved in the regulation of OCSCs stemless by ST6GALNAC1. When compared with the NC group, the mRNA and protein levels of CD44, Nanog, Oct4, CXCL16, and EGFR (Fig. 8a, b), and the rate of EdU-positive cells, abilities of cell migration, invasion, tumorsphere formation and colony formation were all significantly elevated (Fig. 8c, f) in the ST6GALNAC1 + DMSO group, which were all prevented after LY294002 treatment. Therefore, inhibiting the Akt signaling pathway could reverse the effect of ST6GALNAC1 in promoting the proliferation, migration and invasion of OCSCs, as well as the acquisition of stemless of OCSCs.

### Inactivation of the Akt signaling pathway suppressed in vivo tumorigenicity of OCSCs

**Fig. 9:**
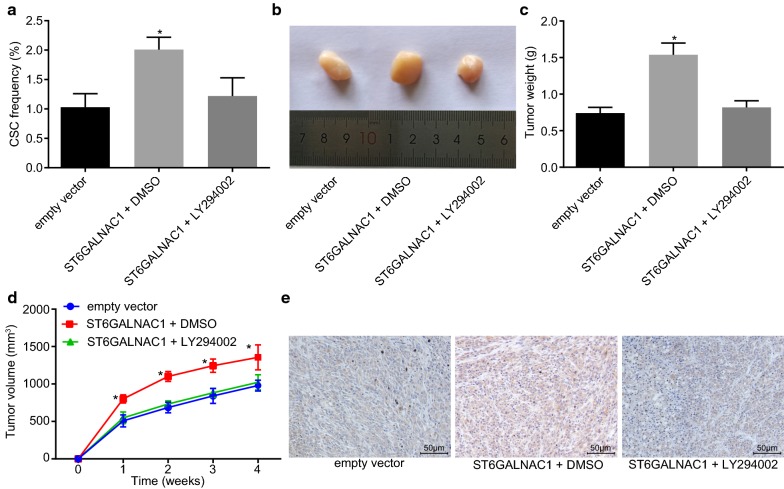
Inactivated Akt signaling pathway inhibits in vivo tumorigenicity and tumor growth of OCSCs. **a** In vivo limiting dilution assay was used to detect in vivo stem cell percentage of ST6GALNAC1 in mice; **b** after transfection, the tumor size of different cells in nude mice was determined; **c** tumorigenic tumor weight in nude mice; **d** changes of tumor volume in nude mice; **e** β-catenin staining intensity in OCSCs detected by immunohistochemical staining (×200). The measurement data were expressed as mean ± standard deviation. The in vivo stem cell ratio of ST6GALNAC1 in mice and quantification of tumor volumes were analyzed by one-way ANOVA. The quantification of tumor weights was examined by repeated-measurement ANOVA (n = 6). *p < 0.05 vs. the empty vector group; the experiment was repeated 3 times

In order to identify the roles of the Akt signaling pathway in tumorigenicity of OCSCs, in vivo limiting dilution experiments were performed. When compared with the empty vector group, the number of tumors and the proportion of cancer stem cells formed in the ST6GALNAC1 + DMSO group were higher, which were both reduced in the ST6GALNAC1 + LY294002 group (Table 4, Fig. 9a). Mouse subcutaneous tumor model also showed that when compared with the empty vector group, the ST6GALNAC1 + DMSO group exhibited earlier tumor formation, faster tumor growth, and larger tumor volume, which had not been seen in the ST6GALNAC1 + LY294002 group (Fig. 9b, d). As shown by immunohistochemical staining, compared with the empty vector, the ST6GALNAC1 + DMSO group had promoted β-catenin staining intensity. However, with the addition of LY294002, the β-catenin staining intensity was reduced (Fig. 9e). Therefore, ST6GALNAC1 induced tumor growth of OCSCs could be suppressed by inactivation of the Akt signaling pathway.

**Table 4:** Tumorigenicity of CD90+ spheroid cells

Injected cells	Empty vector	ST6GALNAC1 + DMSO	ST6GALNAC1 + LY294002
1000	0/5	1/5	0/5
5000	1/5	2/5	1/5
10,000	2/5	3/5	3/5
50,000	3/5	5/5	3/5
Total	6/20	11/20	7/20

DMSO, dimethylsulfoxide

## Discussion

**Fig. 10:**
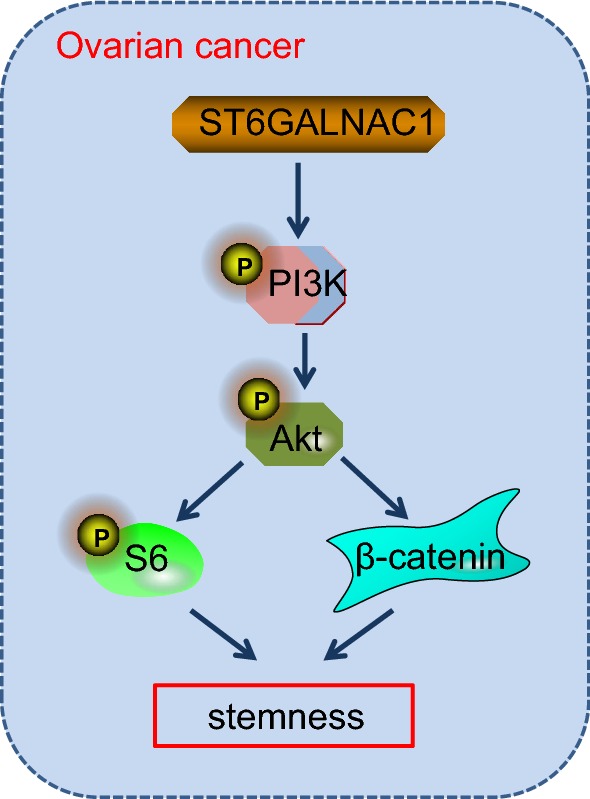
Activation of the Akt signaling pathway by ST6GALNAC1 enables tumor cells to acquire stem cell stemness. ST6GALNAC1 promotes the phosphorylation of PI3K, Akt, and S6 and increases β-catenin transcription to activate the Akt signaling pathway and finally enables tumor cells to acquire stem cell stemness

ST6GALNAC1 had the ability to encode a sialytransferase that acts as a catalyst for production of the cancer-related sTn, which is expressed in a range of carcinomas and related with metastasis and poor prognosis in patients with cancer [11]. For instance, overexpression of ST6GALNAC1 in a gastric cancer cell line enhanced metastatic ability and tumor growth [19]. ST6GALNAC1-overexpressed breast cancer cell lines also exhibited enhanced tumorigenicity [20]. Mechanically, ST6GALNAC1 had been demonstrated to be a potential participator of miR-370 in OCSCs and tumor cells. The CSC assumption suggests that tumors are maintained by a subpopulation of cells with stem cell features [21], which express pluripotent genes and stem cell markers such as Nanog and Oct4. Furthermore, Oct4 and Nanog have shown to be highly expressed in side population cells obtained from ovarian cancer cell lines, which reveal the expression of stem cell markers in ovarian cancer [22]. Stem cell markers have the main function of controlling the embryonal stem cells self-renewal and differentiation and maintaining the biological characteristics [23]. CD44 is a hyaluronic acid receptor and has been found to participate in diverse cellular processes, such as tumor metastasis, cell migration, proliferation [24]. Its elevation has been shown to be correlated to poor prognosis of numerous cancer types [25]. Furthermore, CD44 could serve as a promising biomarker to predict the diagnosis and prognosis of patients suffering from ovarian cancer [26]. In this current study, it was shown that ST6GALNAC1 was overexpressed in ovarian cancer cell lines and OCSCs [27]. It was also found that a reduced expression of CD44, Nanog, Oct4, CXCL16 and EGFR after ST6GALNAC1 silencing in OCSCs indicated that CD90^+^ stem cells infected with miRNA against ST6GALNAC1 had weaker self-renewal capacity in ovarian cancer (Fig. 10).

In breast and colon cancer, overexpression of ST6GALNAC1 had been shown to increase tumor growth, migration and metastatic abilities [20]. Moreover, inhibition of ST6GALNAC1 resulted in lower sphere-forming ability, indicating the maintenance effects of ST6GALNAC1 on cancer cells [4]. In this study, it was observed that overexpression of ST6GALNAC1 promoted proliferation, invasion and migration abilities of OCSCs, together with increased expression CXCL16 and EGFR in OCSCs. The silencing of ST6GALNAC1 exhibited opposite effects, suggesting that ST6GALNAC1 silencing could confer inhibitory effect on OCSCs proliferation, invasion and migration. CXCL16, deemed as a unique chemokine, exists both in transmembrane or soluble forms, and the CXCL16/CXCR6 chemokine axis is recently occupying roles in cancer as regulators of cell proliferation, invasion and metastases [28]. More importantly, the expression of CXCL16 and CXCR6 had been implied to be overexpressed in epithelial ovarian cancer which was closely associated with tumor growth, proliferation, invasion and lymph node metastases [29]. EGFR overexpression has also been demonstrated to play a major part in cancer progression, especially in tumor invasiveness and aggressiveness [30]. Overexpression of EGFR is frequently occurred in 30% to 98% of epithelial ovarian cancer; apart from that, intracellular EGFR dimerization could activate signaling pathways including the PI3K/Akt signaling pathway which functions as a regulatory role in tumor migration and invasion, cellular functions, as well as resistance to cell apoptosis in epithelial ovarian cancer [31].

Previous studies have also found that the Akt signaling pathway was activated by ST6GALNAC1 via galectin-3 [4]. It was also proven that in OCSCs, ST6GALNAC1 in fact promoted the biological characteristics, cell proliferation, and migration and invasion abilities by activating the Akt signaling pathway. The Akt signaling pathway is one of the most important pathways to be associated with cell proliferation [32]. Accumulating studies reveal that the Akt signaling pathway has played an important part in promoting the emergence, progression, invasion and metastasis of cancers, such as hepatocellular cancer [33]. The Akt signaling pathway has been confirmed as a carcinogenic pathway in ovarian cancer [34]. Furthermore, the Akt signaling pathway may mediate miR-93-induced chemoresistance to cisplatin in A2780 cells, providing a novel approach to the study of drug resistance and may contribute to discovering new therapies curing ovarian cancer [35]. A study has suggested that high expression of ST6GALNAC1 is found in colon carcinogenic tumor stem cells, and high expression of ST6GALNAC1 can promote the expression of tumor stem cell marker protein CD44-associated antigen Stn, which can not only promote the tumorsphere-forming ability of tumor cells, but also promotes the resistance of tumor cells to chemotherapeutic drugs [4]. Based on the analysis, it was found that the Akt signaling pathway was active in tumor cells with high expression of ST6GALNAC1, and the activation of the Akt signaling pathway could be blocked by knock downing galectin-3 protein. It can be concluded that ST6GALNAC1 cooperates with galectin-3 protein to activate the Akt signaling pathway. Meanwhile, a study has demonstrated that silencing galectin-3 can inhibit the migration and invasion of pancreatic cancer cells by down-regulating the level of Akt phosphorylation and GSK-3β protein [36]. Another study has indicated that the use of galectin-3 inhibitors can block the binding of galectin-3 to EGFR, thus inhibit the galectin-3/EGFR/Akt/FOXO3 axis and inhibit the occurrence and development of pancreatic cancer in vivo and in vitro [37]. Therefore, it can be concluded that ST6GALNAC1 promotes the activation of EGFR/Akt/FOXO3 signaling pathway by synergetic effect with galectin-3. The specific mechanism needs further verification. In the future, it could be inferred that the Akt signaling pathway may play an important part in the biological features of OCSCs.

## Conclusion

In conclusion, the present study suggests that ST6GALNAC1 activates the biological characteristics of OCSCs in ovarian cancer, which is achieved through increased proliferation, invasion and migration of OCSCs via activation of the Akt signaling pathway (Fig. 9). These findings may open novel avenues for future ovarian cancer therapies. However, further study is still needed to better understand the underlying mechanisms of ST6GALNAC1 regulating the Akt signaling pathway in ovarian cancer.

## Abbreviations

OCSCs: ovarian cancer stem cells
CICs: cancer-initiating cells
GEO: gene expression omnibus
CD90+: cluster of differentiation90+
RT-qPCR: reverse transcription quantitative polymerase chain reaction
GAPDH: glyceraldehyde 3-phosphate dehydrogenase
SDS-PAGE: sodium dodecyl sulfate polyacrylamide gel electrophoresis
PVDF: polyvinylidene fluoride
DMEM: Dulbecco’s Modified Eagle Medium
EGF: epidermal growth factor
FOLR1: folate receptor
